# *In vivo* evaluation of the toxic activity and genotoxicity of the *Hymenaea courbaril* L.’s resin in *Drosophila melanogaster*

**DOI:** 10.1016/j.sjbs.2021.09.005

**Published:** 2021-09-13

**Authors:** Jorge Anaya-Gil, Patricia Ramos-Morales, Adriana Muñoz-Hernandez, Adriana Bermúdez, Harold Gomez-Estrada

**Affiliations:** a Natural Products Group (Grupo de Productos Naturales), School of Pharmaceutical Sciences, Zaragocilla Campus, University of Cartagena, Cartagena 130015, Colombia; b Laboratory of Genetics and Environmental Toxicology – Banco de Moscas, National Autonomous University of Mexico. Mexico; c Descriptive and Applied Biology (Biología descriptive y aplicada) Research Group. University of Cartagena, Cartagena, Colombia; d Medicinal Organic Chemistry Research Group (Grupo de Química Orgánica Medicinal), University of Cartagena, Cartagena, Colombia

**Keywords:** *Hymenaea courbaril* L., Resin, genotoxicity, *Drosophila melanogaster*, SMART

## Abstract

Due to the negative consequences carried by the usage of synthetic insecticides, a global interest into finding substitutes for these chemical compounds through natural products has arisen. When yielded to external attacks, plants generally produce metabolites to defend themselves. The physicochemical characteristics of this kind of compounds have allowed their usage as potential bioinsecticides. The *Hymenaea courbaril* L. (algarrobo) has proven to be a plant rich in metabolites with outstanding biological activity, in such a way that some of its extracts have been tested as insecticides. The goal of this study was to know the phytochemical composition of *Hymenaea courbaril* L.’s resin and perform evaluations *in vivo* of its toxic and genotoxic effects in the biological model *Drosophila melanogaster*. For this, two resin extracts were prepared and both a phytochemical analysis were carried out on them, having found in the ethanolic total extract the presence of terpenes, flavonoids and coumarins, while in the partial ethanolic extract only presence of terpenes and flavonoids was found. *Drosophila* larvae were submitted to different concentrations of the extracts and both the survival and the sexual ratio were evaluated, finding that larvae are more sensitive to the partial ethanolic extract. Subsequently, the induction of somatic mutation and mitotic recombination (SMART) was evaluated in the flies’ eyes. The most significant affectations at a genotoxic level were found when larvae were tested with the partial extract, indicating that possibly the coumarins absence makes this insect more susceptible to damages at a genetic material level.

## 1 Introduction

Colombia is an agricultural country par excellence, whereby the pesticides usage has become a basic need in farming, to the end of controlling diseases, insects, weeds, and other organisms interfering with agricultural production (Badii and Varela, 2015). For insects control, pesticides of chemical synthesis are mainly used (Sarwar, 2016); however, the extended usage of this kind of compounds has been associated with negative consequences, among which are: increasing contamination of water bodies, soil and air pollution, increased level of toxicity, natural protection systems weakening because of the negative impact on organisms beneficial to the ecosystems, resistance generation in different pests who survived to this exposure and also human health alterations (Simon-Delso et al., 2015, Chen et al., 2019, Yun et al., 2017). Human beings are exposed to pesticides through different routes such as inhalation, ingestion and dermal contact and this can lead to acute and chronic health issues (Zanabria et al., 2019).

To overcome this problem, bioinsecticides usage has been proposed as an alternative which have such advantages as high biodegradability, low toxicity (Kumar and Singh, 2015) and an ample structural diversity that can delay resistance generation. A promising natural pesticides source are the plants-extracted metabolites (Mathew, 2016). Because of having evolved simultaneously with insects, these plants have developed diverse defense mechanisms, which can be either of physical or chemical type, that have favored its adaptation. Among the chemical ones there is the insecticidal action o repellent (Sisay et al., 2019), which has been used by mankind as a tool to fight plagues. Unlike animals, plants have not a circulating immune system to recognize microbial pathogens. Plant cells are more autonomous in its defense mechanisms, which are based both in the innate immune capacity of each cell and in systemic signals propagated from the infection sites (Liu and Coaker, 2008). A large number of investigations have been centered in the interactions between secondary metabolites and insect plagues attacking plants, particularly those causing economic damages, in order to find natural bioactive insect-specific products that allow the designing of rational and sustainable methodologies with low environmental impact, to control them (Jaramillo-Colorado et al., 2015).

For this purpose and taking advantage of the plant species diversity present in the Caribbean Colombian Region, the *Hymenaea courbaril* L. was selected as a possible natural source of metabolites with insecticidal capacity. The *Hymenaea* genus (Fabaceae) includes twenty-nine species; twelve which are distributed through South America, five species have been reported in Colombia (*Hymenaea courbaril* L., *Hymenaea intermedia* Ducke, *Hymenaea martiana* Hayne, *Hymenaea oblongifolia* Huber and *Hymenaea parvifolia* Huber) (Lee and Langenheim, 1975, Bandeira et al., 2015, WFO, 2021, Gradstein, 2015). Among these species is *Hymenaea courbaril* L., which is commonly known as carob tree, algarrobo, guapinol, lucust, jutaby or courbaril (Alzate Tamayo et al., 2008). It is an imposing forest tree producing large and very tough sheaths containing a penetrating smelling pulp, but edible, and large seeds used to animal and human feeding. *Hymenaea* genus species are source of a variety of active secondary metabolites (Fernandes et al., 2005).

The phytochemical analysis of various parts of the plant shows the presence of flavonoids, oligosaccharides, sesquiterpenes, diterpenes and phenolic compounds (Dias et al., 2013). When submitted to mechanical stress or animal attack, this plant is characterized for producing a resinous exudate (Schwartz, 2018), which provides it both a chemical and mechanical protection (Langenheim, 2003). *Hymenaea courbaril* L. resin is rich in metabolites with different physical–chemical characteristics (Braga et al., 2000, Gonçalves et al., 2005, Jayaprakasam et al., 2007) which turn it into a promising substance to study the possible applications of some of its components as insecticide.

To test the insecticidal ability of the *Hymenaea* resinous exudate and some of its components, it was decided to use the model organism *Drosophila melanogaster* (De Morais et al., 2017, Oliveira et al., 2020, Senthilkumar et al., 2020). This dipteran, which has been used in numerous investigations, has a life-cycle relatively short (10 days at 25 °C) (Liu et al., 2019), but long enough to perform *in vivo* trials of acute and chronic doses. This insect also provides large samples and the toxic effects can be seen through many generations, in a relatively short period of time (Botas, 2007). It has been successfully used to verify the toxic effects of natural substances from various plants (Panchal and Tiwari, 2017). Additionally, this insect has a high genetic homology with superior organisms (Vecchio et al., 2012, Zhou et al., 2018). This dipteran could complement the insecticidal activity search in the resin components, besides alerting us about the capacity of the latter to damage the hereditary material.

Among the diverse methodologies developed with *Drosophila melanogaster*, the somatic mutation and mitotic recombination test in the eyes (SMART) is an efficient alternative to indicate occurring changes in the hereditary material of the exposed organisms. It is based on the heterozygosity loss in the larvae (*w/w^±^*) exposed to the substance of interest, which can occur by mutation, deletion or nondisjunction, but mainly by mitotic recombination (95 % of cases) (Vogel and Zijlstra, 1987, Lasko et al., 1991, Palermo and Mudry, 2014). This investigation goal was to phytochemically characterize *Hymenaea courbaril* L. resin and then evaluate the toxic and genotoxic activity of some of its components in *Drosophila melanogaster.*

## 2 Materials and methods

### 2.1 Plant material and extraction

*Hymenaea courbaril* L. resin was collected in Simití-Bolívar (Colombia), in December 2017. The specimen registration is available at the University of Antioquia’s herbarium (Colombia) (voucher HUA 214538).

#### 2.1.1 Extraction

Unless otherwise stated, all the chemical reactives acquired (Merck o Aldrich) were of the highest purity commercially available and they were used with no previous purification. An ethanolic total extract was obtained by putting 5 g of *Hymenaea courbaril* L. resin powder in 15 mL of ethanol (from now on, total extract). The mixture was subjected to magnetic stirring at 300 rpm for 18 h and then to sonication for 2 h. After that, cannula filtration was performed and the previous stages were repeated three more times to get the total volume of 60 mL. Each extraction was monitored by Thin Layer Chromatography (TLC) (Kumar et al., 2013, Sosa et al., 2019). To conclude, the final extract total volume was concentrated in a rotary evaporator.

A preliminary phytochemical screening of the total extract was performed to determine the secondary metabolites presence using TLC, to later have the layers revealing the metabolites considered as promising according to the bibliographic review made: triterpenes and steroids (Salkowski test), flavonoids (citroboric acid test), coumarins (KOH test in ethanol) and alkaloids (Draguendorff test) (Santos et al., 2017).

**Fig. 1:**
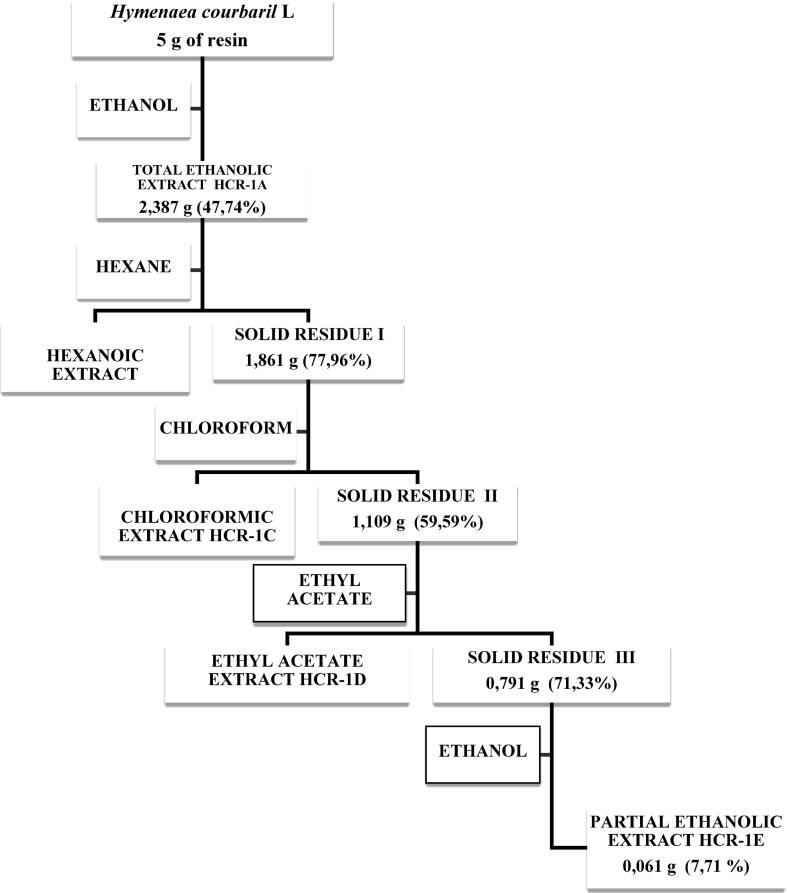
Scheme of preparation of *Hymenaea courbaril* L. resin extracts.

From the total extract a partial extract was obtained as follows: 5 g of total extract were solubilized in 20 mL of hexane and were sonicated for 2 h. The insoluble part in hexane was dissolved in chloroform with the same procedure. In the same way, the undissolved part of this last stage is dissolved in ethyl acetate and the insoluble residual part was, again, dissolved in ethanol which was denominated as partial ethanolic extract (from now on, partial extract), to which it was performed the same phytochemical analysis procedure and it was also used in the biological trials. Fig. 1 shows the extraction scheme with the obtained results for both the total extract and the partial extract.

In order to identify some chemical compounds present in the resin, it was performed an approximate characterization through gas chromatography coupled to mass spectrometry, under analysis conditions described in the next section.

### 2.2 Gas chromatography–mass spectrometry (GC–MS)

Experiments were carried out with an Agilent Technologies 7890A, gas chromatograph equipped with an on-column injection system and coupled with an Agilent Technology 5975 mass spectrometer. It was used an HP-5 capillary column (5% phenyl – 95% methyl siloxane, 30 m × 250 µm × 0.25 µm film thickness).

The chromatographic conditions were as follow: a GC temperature of initially 70 °C the programed at 10 °C min^−1^ to 150 °C for 1 min, then 8°Cmin^−1^ to 208 °C for 1 min. The inlet was set at 70 °C. Samples were injected in the split mode (10:1 split ratio). The electronic pressure control was set to the constant flow mode with vacuum compensation. The helium gas flow was set at 1 mL min^−1^.

Ions were generated by electron impact (EI) ionization (electron energy 70 eV) in the ionization chamber of the mass spectrometer. The mass spectrometer was scanned from *m*/*z* 30 to 700, with a cycle time of 1 s. The Agilent ChemStation software G1701CA MSD was used for GC–MS control, peak integration and mass spectra evaluation. The tuning of the mass spectrometer was carried out using dichloromethane. The temperatures of the interface and the source were 280 and 150 °C, respectively. Compounds were identified by using NIST and Wiley mass spectral libraries.

After the identification of some of the metabolites present, biological assays were performed.

### 2.3 *Drosophila* as a test system

#### 2.3.1 Somatic mutation and mitotic recombination test (SMART)

During larval development, cells that will originate the eye structures multiply by mitosis. These are genetically determined but they differentiate until metamorphosis. In adult flies, each ommatidia is formed by 24 cells, two of which are the primary pigmented ones that physically isolate one ommatidia from another and in which are deposited the pigments (ommochromes and pterins) that give flies eyes color. If during larval development there is heterozygosity loss in some of the precursor cells that will originate the eyes, this modification will be passed on to daughter cells through mitosis and on the adult fly primary pigmented cells will lack pigment and form a mutant spot (*w*) in the adult fly eye.

To evaluate the mutation and somatic recombination induction, the number and size of the recovered spots in each eye were registered. By anesthesia excess, flies are sacrificed and are placed on an excavated plate in a 90% ethanol, 1% tween 80 and 9% water solution. Eyes spots are registered with the assistance of a stereo microscope, with 120x of augmentation (Ramos-Morales et al., 2001). Two spots are considered independent if they are separated by 4 or more non affected ommatidia rows (Vogel et al., 1985).

#### 2.3.2 Biological material

The strains used were provided by the *Drosophila* Stock Center of the Sciences Faculty, National Autonomous University of Mexico. The cultures were kept at constant temperature and humidity (25 ± 1 °C; 60% of relative humidity, respectively) on *Drosophila* medium comprising of agar (0.56 %), cornflour (5.9%), sugar (7.6%), beer yeast (4%), nipagin (methyl-p-hydroxybenzoate) (0.22%), propionic acid (0.22%) and water (81.4%).

*White, w* (1–1.5), recessive mutation, linked to the sex, flies’ eyes are white. From this strain, females were used (Lindsley and Zimm, 1992).

Wild, (*Canton S*), flies without known mutations or specific arrangements in their chromosomes, eyes are red. From this strain, males were obtained for the experimental cross and it was also used as a reference cross.

Experimental cross: virgin females, *w/w* × wild males (*w^±^/Y*). In the progeny, the *w/w^±^* females will have red eyes and (*w/Y*) males show white eyes, so the latter are only used for toxicity assessment.

#### 2.3.3 Egg collection and obtaining larvae

Three days after both experimental cross and wild cross were carried on, flies are transferred to fresh medium jars for them to oviposit in an 8 h period. After that, progenitors were retired and the eggs were left to continue their development until reaching larvae stage of third instar (72 ± 4 h age). Larvae are extracted from the medium using a 20% sucrose solution which make them float by density difference (Nöthiger, 1970). Solution with larvae is thrown into a 1 L separation funnel, with a 3.4 mm diameter pore. They are washed with tap water to remove sucrose traces and are recovered in a net. Groups of approximately 100 larvae are placed in each tube, which contains 1 g of instant medium for flies Carolina® (Formula 4–24 Plain, Carolina Biological Supply Company) and 4.5 mL of the solution to be tested. Larvae were fed with this medium, until metamorphosis was induced (48 h) and thereafter they emerged as adult flies.

### 2.4 Treatments

#### 2.4.1 Toxicity assessment

A toxicity-concentration curve was made, which included 13 concentrations into a 50 ppm to 2.05 E−07 ppm range, which were obtained through successive dilutions from a stock of 50 ppm of the *Hymenaea courbaril* L.’s resin total extract, and 13 concentrations in a 19.1 ppm to 7.86E−08 ppm range for the partial extract. Due to samples low solubility in water, a 2% ethanol solution was used as solvent and as a negative control. For each tested concentration, two replicas were made per experiment, and the whole experiment was repeated three times.

Treatment toxicity was estimated through the survival mean per vial, compared with the control batch survival through a simple two-tailed ANOVA (α = 0.05), followed by a Dunnett test (α = 0.05), in case of significant differences.

It was determined if the treatment affects preferentially females or males through the proportions of flies of one sex recovered at each concentration. In the present work, the ratio between the number of females with respect to the total number of females and males in each vial (sex ratio) was calculated. The average sex ratio per concentration from the replicates of each concentration into an experiment and from the three experiments was obtained.

The effect of treatment on sex ratio in the control and experimental series was compared through the total sum of recovered flies in the three experiments and a Z-test for ratios was done (α = 0.05).

#### 2.4.2 Total and partial extract genotoxicity determination of *Hymenaea courbaril* L.’s resin

Somatic mutation and mitotic recombination frequency was estimated as the number of spots per 100 eyes. The frequency of spots in the experimental and control series was compared through a Z-test for ratios, with α = 0.05.

To graphically compare the induction of somatic mutation and mitotic recombination of the adult flies recovered from the treatments with total extract and partial extract, frequency of mutant spots of each of the controls was subtracted from the control and experimental series corresponding to each treatment. Thus, both control groups remain with a frequency = 0 (corrected frequency of spots per eye).

## 3 Results

### 3.1 Total extract and partial extract of *Hymenaea courbaril* L.’s resin

To obtain the different extracts, techniques favoring metabolites extraction presents in the resin were used, including the sonication process, which has gained great momentum when it was found that it allows to increase the solubility of the biologically active components (López et al., 2016), with good performance and high purity. The metabolites extraction present in the resin was carried on exhaustively in order to obtain the total extract with as many metabolites as possible. Four consecutive extractions were enough to ensure the extraction of the metabolites present in the resin. The extraction process follow up was performed through Thin Layer Chromatography (TLC) (Sosa et al., 2019). The phytochemical analysis (Table 1) revealed that the metabolites found in the total extract correspond with what has been reported in this kind of materials, like terpenes presence (Nogueira et al., 2001) and phenolic compounds presence (De Sousa et al., 2020), to which insecticidal action is attributed (Fernandes et al., 2005). In contrast, in the partial extract coumarins presence was not detected.

**Table 1:** Phytochemical screening of the total extract and ethanolic fraction of resin of *Hymenaea courbaril* L.

Constituents	Test performed	Total extract	Partial Ethanolic Extract
Terpenoids and steroids	Salkowski’s Test	**+**	**+**
Flavonoids	Citroboric reagent test	**+**	**+**
Coumarins	KOH in ethanol test	**+**	**−**
Alkaloids	Draguendorff́s test	**−**	**−**

**+**: Present; **−**: Not detected.

In order to identify some chemical compounds and verify the presence of biologically active metabolites in the *Hymenaea courbaril* L*.*’s resin, an analysis through gas chromatography coupled to mass spectrometry was conducted. The majority of the compounds identified in the resin are sesquiterpenes (*trans*-α-bergamotene, nerolidol, α-selinine, epicubenol) and diterpenes (kolavelool, copalol, kolavenol). Particularly, the presence of α-copaene, caryophyllene, caryophyllene oxide, α-curcumene, α-humulene, β-bisabolene stands out, which has been reported by McCoy et al. (2017). Additionally, the presence of the flavonoid quercetin and 6-nitrocoumarin was identified. None of the identified peaks matched with some type of alkaloid, which indicates that this analysis is in accordance with the phytochemical screening made in this work. Various of the identified metabolites have been associated with various types of biological activity.

### 3.2 *Hymenaea courbaril* L.’s toxicity in *Drosophila melanogaster*

#### 3.2.1 Survival (S) and sexual ratio (SRx)

In Table 2a is presented the total number of recovered flies, the mean of flies per vial and the mean sexual ratio of females obtained after the treatment with the total extract of *H. courbaril* L.’s resin, which proved not to be toxic for the fly at these concentrations because it was recovered an average of flies similar to that of control group and even lightly superior, except for the last tested concentration (50 ppm) in which the survival was of only 2.67 ± 1.17 flies per vial (p < 0.05).

**Table 2:** Flies recovered after larval treatment with a) Total Extract or b) Partial Extract of *H. courbaril* L. resin.

		Flies/vial		Females/vial	
[ppm]	N	Average ± SE	d^a^	Average ± SE	d^b^
*a) Total Extract*					
OH 2%	436	72.67 ± 8.86		0.48 ± 0.05	
2.05E−07	483	80.50 ± 9.11		0.54 ± 0.02	
1.02E−06	456	76.00 ± 9.90		0.55 ± 0.02	
5.12E−06	444	74.00 ± 7.69		0.57 ± 0.03	
2.56E−05	433	72.17 ± 7.15		0.55 ± 0.03	
1.28E−04	374	62.33 ± 5.39		0.47 ± 0.04	
6.40E−04	502	83.67 ± 10.95		0.55 ± 0.01	
3.20E−03	503	83.83 ± 6.28		0.51 ± 0.02	
1.60E−02	452	75.33 ± 6.94		0.53 ± 0.03	
8.00E−02	432	72.00 ± 8.13		0.49 ± 0.03	
4.00E−01	390	65.00 ± 6.59		0.52 ± 0.04	
2.00	388	64.67 ± 6.84		0.5 ± 0.03	
10.0	338	56.33 ± 4.77		0.57 ± 0.02	
50.0	16	2.67 ± 1.17	*	0.48 ± 0.15	*

*b) Partial extract*					
OH 2%	644	107.33 ± 14.63		0.48 ± 0.02	
7.86E−08	591	98.50 ± 9.08		0.50 ± 0.03	
3.93E−07	594	99.00 ± 9.83		0.51 ± 0.02	
1.97E−06	445	89.00 ± 5.24		0.51 ± 0.02	
9.83E−06	469	78.17 ± 8.70		0.51 ± 0.02	
4.91E−05	547	91.17 ± 5.26		0.50 ± 0.02	
2.45E−04	458	76.33 ± 5.32		0.47 ± 0.03	
1.22E−03	487	81.17 ± 7.81		0.51 ± 0.03	
6.14E−03	482	80.33 ± 4.51		0.52 ± 0.01	
3.07E−02	524	87.33 ± 7.67		0.50 ± 0.02	
1.54E−01	554	92.33 ± 8.37		0.49 ± 0.03	
7.68E−01	533	88.83 ± 4.90		0.47 ± 0.02	
3.84	539	89.83 ± 6.54		0.49 ± 0.03	
19.20	14	2.33 ± 0.92	*	0.82 ± 0.08	*

SE, standard error; d, Statistical diagnosis using: ^a^, ANOVA, two tails, p < 0.05, followed by Dunnett test, p < 0.05; ^b^,  test for proportions, p < 0.05.

In contrast, the flies survival treated with the partial extract was lower in all treatments, although only in the higher tested concentration (2.33 ± 0.92) (p < 0.05) (Table 2b) significant differences were confirmed. In the comparison, the partial extract treatment turned out to be more toxic for *D. melanogaster*.

In regards with sexes ratio on the recovered flies, only in the highest tested concentration on both extracts significant differences were confirmed (p < 0.05). However, while comparing the frequency of recovered females in the total extract, a larger fluctuation was found in comparison with the more homogenous response to the treatment with the partial extract of *H. courbaril* L.’s resin (Tables 2a and b).

The recovered sexes ratio from the treated flies during the larval stage informs about whether some sex is more affected than the other after the same treatment. Tables 2a and b show female ratio versus each one of the extracts concentration. The obtained results do not show a preferential effect either on females or males, but it is noticeable that there is more variability in the mean female ratio when the third stage larvae were exposed to the total extract.

#### 3.2.2 *Hymenaea courbaril* L.’s resin genotoxicity in the somatic mutation and mitotic recombination test (SMART)

In plants, insecticidal activity can rely on one or various metabolites and it can also be modulated by others. Some metabolites act as enhancers of the toxic activity and others as inhibitors of it, wherefore in this work the toxicity of the total and partial extracts on *Drosophila melanogaster* was compared. Additionally, it was determined whether both extracts can interfere with the hereditary material of organisms exposed to it.

With the aim to evaluate the genotoxic capacity of the total extract and partial extract of *Hymenaea courbaril* L.’s resin, third stage larvae, heterozygous *w/w^±^,* were sub-chronically fed with cultured medium enriched with concentrations of both extracts, separately. If during larvae stage there is heterozygosity loss this could lead to mutant spots formed by no pigmented ommatidia. The size of the mutant spots suggests whether the event which originated it happened close to the treatment administration (big spots) or close to the metamorphosis, at the end of the cells proliferative activity (small spots).

To compare clearer the present activity in both total and partial extracts, the concentrations of the total extract will be presented based on the performance obtained after the metabolites extraction. Per 2.387 g of total extract 0.061 g of partial extract were obtained, therefore the highest concentration tested of total extract (50 ppm) would be equivalent to 1.28 ppm of the partial extract. Table 3 shows the equivalent concentrations, which will be used from now on.

**Table 3:** Concentrations of total extract of *H. courbaril* L. used and corresponding concentrations based on the partial extract yield.*

Total Extract [ppm]	Partial extract [ppm]
2.05E−07	5.23E−09
1.02E−06	2.62E−08
5.12E−06	1.31E−07
2.56E−05	6.54E−07
1.28E−04	3.27E−06
6.40E−04	1.64E−05
3.20E−03	8.18E−05
1.60E−02	4.09E−04
8.00E−02	2.04E−03
4.00E−01	1.02E−02
2.00E+00	5.11E−02
1.00E+01	2.56E−01
5.00E+01	1.28E+00

* For every 2.387 g of total extract, 0.061 g of partial extract is obtained.

Table 4 shows the results obtained for the SMART test. The treatment with the total extract increased eyes mutant spots frequency from the lowest tested concentration and up to 2.04E-03 ppm (p < 0.05), with the exceptions of 1.31E-07, 3.27E-06, 4.09E-04 concentrations and the four highest concentrations; wherefore it is important to write down that a linear response between the administered concentration and mutant spot frequency was not found (Table 4a).

**Table 4:** Spots per eye from flies treated with total extract or partial extract of *H. courbaril* L. resin.

[ppm]	N	n	Spots/eye	d^a^
*a) Total extract*				
0/ OH 2%	320	26	(0.081)	
5.23E−09	250	37	(0.148)	*
2.62E−08	250	34	(0.136)	*
1.31E−07	250	32	(0.128)	
6.54E−07	239	32	(0.134)	*
3.27E−06	178	19	(0.107)	
1.64E−05	250	35	(0.140)	*
8.18E−05	254	35	(0.138)	*
4.09E−04	243	30	(0.123)	
2.04E−03	212	30	(0.142)	*
1.02E−02	203	19	(0.094)	
5.11E−02	196	17	(0.087)	
2.56E−01	195	18	(0.092)	
1.28E + 00	7	0	0	

*b) Partial extract*				
OH 2%	320	26	(0.081)	
7.86E−08	250	30	(0.120)	
3.93E−07	250	48	(0.192)	*
1.97E−06	231	40	(0.173)	*
9.83E−06	239	56	(0.234)	*
4.91E−05	250	47	(0.188)	*
2.45E−04	223	32	(0.143)	*
1.22E−03	250	16	(0.064)	
6.14E−03	250	38	(0.152)	*
3.07E−02	250	40	(0.160)	*
1.54E−01	250	35	(0.140)	*
7.68E−01	250	27	(0.108)	
3.84E + 00	250	47	(0.188)	*
1.92E + 01	10	2	(0.200)	

N, number of flies: n number of spots; d^a^, Statistical diagnosis using  test for proportions, two tails, p < 0.05.

The treatment with the partial extract showed greater genotoxic activity (Table 4b) (p < 0.05), although the response was not linear either. Just in the 7.86E−08, 1.23E−03 and 7.68E−01 concentrations the frequency was similar to that of the control. In the highest concentration tested, although the mutant spots frequency increased, the sample size retrieved was not enough to establish a statistical diagnosis.

**Fig. 2:**
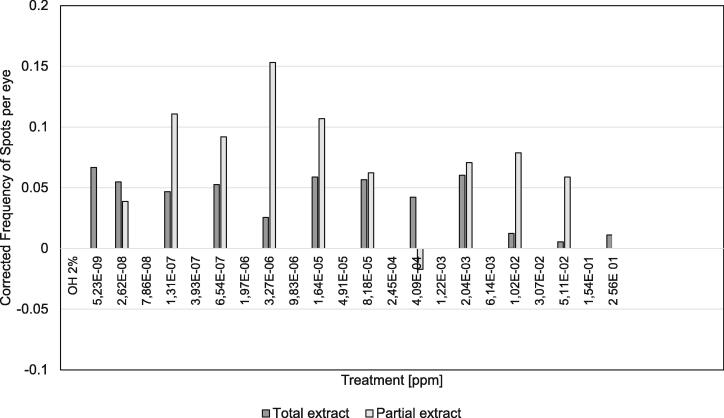
Corrected Frequency of Spots per eye from flies treated with total extract or partial extract of *H. courbaril* L. resin.

In Fig. 2 is compared the corrected mutant spots frequency in the eyes obtained from both treatments. Despite having found genotoxic activity in both extracts, the increment of the mutant spots frequency in the eyes of *D. melanogaster* was higher when the partial extract was used (clearer bars).

## 4 Discussion

With the idea to find promising metabolites as potential insecticides obtained from plants, in the current research it was opted to characterize chemically *Hymenaea courbaril* L.’s resin, which was submitted to metabolites extraction processes using ultrasound, since it has been reported that it offers advantages as extraction method, both used individually and combined with other clean and sustainable technologies, in regards to traditional methods. The greatest achievements are based in the reduction of the extraction time and the increase of both efficiency and purity of the extracted compound (Rodríguez-Riera et al., 2014), in such a way that by using the sonication it is tried to guarantee the recovery of as much as possible metabolites at the moment of preparing an extract. In Fig. 1 is shown that starting with 5 g of resin, 2.387 g of total extract are obtained and when performing the successive extractions to obtain the partial extract, 0.061 g of it are obtained. This difference in the amount of retrieved mass from both extracts is directly related to the metabolite loss in the different extraction processes; for instance, the presence of coumarins in the total extract, which were not detected in the partial extract. Coumarins could have been solubilized in the different solvents used, wherefore they could be discarded throughout the different extraction processes. In contrast, terpenes and flavonoids presence were detected in both extracts.

It has been reported that compounds like terpenes and flavonoids have some sort of biological activity when they are evaluated using biological models. The gas chromatography coupled to mass spectrometry was a tool that allowed the identification of some compounds present in the resin, as in the sesquiterpenes and diterpenes case, which have been associated with larvicidal and repellent activity (Cunningham et al., 1974, Nogueira et al., 2001, Bezerra et al., 2013). Here are some investigations wherein metabolites discovered coincide with the ones found in this work. Gupta et al. (2014) found that when *Hymenaea courbaril* L. is submitted to heat-stress compounds like α-copaene, β-elemene, caryophyllene, α-caryophyllene and α-selene have been detected, which favor thermotolerance. Various of the metabolites identified in the resin were found when studying this species flowers (Marinho et al., 2018). Also, common sesquiterpenes in the ethanolic extract of *Hymenaea’s* fruit were found (Sales et al., 2014). In this sense, Aguiar et al. (2010) report that among the main constituents of the essential oil of the ripe fruits peel were: α-copaene (11.1%), and β-selene (8.2%), while β-caryophyllene (27.1%) was found in the oil of immature fruits. These oils were tested for larvicide activity against *Aedes aegypti*. Other metabolites found are: α-copaene, which has shown biological activity as insecticide in *D. melanogaster* (Case et al., 2003); caryophyllene, which is toxic for *Drosophila* in synergy with other metabolites such as sabinene and spatulenol (Bezerra et al., 2017). Other compounds identified in this work, but which have been extracted from *P. guajava*, like α-humulene, caryophyllene and caryophyllene oxide have insecticidal activity on this insect (Pinho et al., 2014). Sesquiterpene nerolidol or peruviol has activity as insect repellent (Chan et al., 2016); α-curcumene has been used as a whitefly repellent in tomatoes (Bleeker et al., 2011). Another molecule with biological activity present in the resin is the kolavenol, with trypanocide activity (Freiburghaus et al., 1998). Hubbell et al. (1983) reported caryophyllene oxide as an important compound in the *Hymenaea* tree antifungal action.

When evaluating the toxicity of plant extracts, it is necessary to remember that the nonlinear responses associated with low concentrations of xenobiotics could be due to the loss of homeostasis in the organism in response to environmental assaults. The adaptive homeostasis could explain both the increase or decrease observed in the homeostatic range of an organism exposed to sublethal conditions (Berry and López-Martínez, 2020). The survival index is a good toxicity biomarker since it shows the *in vivo* organisms response to the xenobiotics presence. In this sense, the *D. melanogaster* larvae were sensitive to extracts composition differences, that is, they responded to metabolites presence or absence as reflected in the amount of retrieved adult flies.

Sexual ratio is a biomarker indicating whether the xenobiotics affect males and females differently. It has been reported that some substances with carcinogenic activity and some endocrine disruptors cause different effects on female and male mammals, mainly associated to the hormonal regulation of the treated animals (Cederroth et al., 2010). Different variability in the recovered female’s ratio in both tested extracts confirms that fly is sensitive to the presence of different metabolites or to different proportion of them.

Studies of plants extracts genotoxicity are necessary, because regardless vegetables derivatives show some biological activity of interest (anthelmintic, insecticidal, antipyretic, etc.), it is necessary to know whether they represent some kind of risk to organisms. Throughout this work, it has been studied the genotoxic potential of the total extract and partial extract of *H. courbaril* L.’s resin, using the Somatic Mutation and Recombination Test (SMART) in the eyes of *D. melanogaster.*

The genotoxic activity evaluation of natural products, and especially of medicinal plants, has become into something essential in order to guarantee long-term risks absence (Amkiss et al., 2021). With SMART methodology, more than 250 compounds have been analyzed (Vogel et al., 1985, Vogel, 1992, Vogel and Nivard, 2000). It has been shown that more than 90% of the genotoxic activity detected by this trial is associated with the mitotic recombination, a post-replicative repair mechanism of the genetic damage (Vogel and Zijlstra, 1987, Sekelsky, 2017), wherefore it is inferred either that in the partial extract there is the presence of metabolites with DNA damage capacity or in the partial extract some metabolites with anti-genotoxic capacity could be eluded or remained in very low concentrations, in such a way that they did not exert the DNA protective effect, as it could be suggested from the coumarins absence.

Terpenes found in the phytochemical analysis of *H. courbaril* L.’s resin are a type of compounds which present different physiochemical properties, among which is the capacity to enhance other toxins effects (Rattan, 2010). It also has been found that some sesquiterpenes have shown to have antitumor potential, for instance α-selinine, which is a functional antioxidant with anti-radical properties (Kiralan et al., 2012); β-bisabolene which has been used for carcinogenic cells treatment (Yeo et al., 2016). Likewise, it has been reported that humulene carcinogenic activity increases in the presence of β-caryophyllene in no-cytotoxic concentrations (Legault and Pichette, 2007). Some flavonoids like quercetin have antioxidant and anticarcinogenic characteristics (Birt et al., 2001, Li et al., 2016).

Successive extractions can remove metabolites, therefore antigenotoxic activity will change, which could partially explain that partial extract presents more genotoxicity. It is important to remember that in the partial extract coumarins presence was not detected, which are a special type of phenolic compounds derivatives with antioxidant capacity. This is due to the product of the existent conjunction between the merged heterocycle which is these molecule base core that favor the electron sequestration from the free radicals (Xi and Liu, 2015). Numerous derivatives of natural coumarins have been studied through, *in vivo* methods, in order to evaluate antioxidant, anticancer and anti-inflammatory activity (Detsi et al., 2017, Katsori and Hadjipavlou-Litina, 2014); it has even been found biscoumarins presence in *Hymenaea courbaril’s* leafs with the capacity to capture free radicals (Simões et al., 2009).

It can be concluded that *Hymenaea courbaril* L.’s resin is rich in terpenoids-type compounds, mainly sesquiterpenes, flavonoids and coumarins. The biological model *D. melanogaster* has demonstrated to have sensitivity to respond differently to the total extract and to the partial extract, which was evidenced in the lower survival retrieved in larvae exposed to the partial extract, which is associated with the toxic activity in coumarins absence. This study confirms the importance of evaluating the genotoxic potential of plants extracts and derivatives for, besides the utilitarian properties they can show, they may represent a risk to organisms exposed to them, especially when having low toxicity, as in the total and partial extracts of *H. courbaril* L.

It is recommended that all studies on the activity of metabolites such as antioxidants, antimutagens or any terminal event of interest be accompanied by determinations of the toxicity of the treatment to avoid confusing a possible protective effect with a toxic effect either at the cellular level or of the whole organism. In this study, *D. melanogaster* was shown to be a reliable model to discriminate between extracts with different composition and to show the *in vivo* effect of complex mixtures such as *H. courbaril* L. resin extracts.

## Declaration of Competing Interest

The authors declare that they have no known competing financial interests or personal relationships that could have appeared to influence the work reported in this paper.
